# Shape analysis of rectus extraocular muscles with age and axial length using anterior segment optical coherence tomography

**DOI:** 10.1371/journal.pone.0243382

**Published:** 2020-12-23

**Authors:** Kiyo Shibata, Atsushi Fujiwara, Ichiro Hamasaki, Takehiro Shimizu, Reika Kono, Keisuke Kanenaga, Masanori Nakazawa, Yuki Morizane

**Affiliations:** 1 Department of Ophthalmology, Okayama University Graduate School of Medicine, Dentistry and Pharmaceutical Sciences, Okayama City, Okayama, Japan; 2 Department of Orthoptics, Faculty of Rehabilitation, Kawasaki University of Medical Welfare, Kurashiki City, Okayama, Japan; 3 Department of Ophthalmology, Kagoshima University Graduate School of Medical and Dental Sciences, Kagoshima, Japan; University of Florida, UNITED STATES

## Abstract

**Purpose:**

This study aimed to evaluate the shape of the extraocular muscles (EOMs) in normal subjects using the en-face images of anterior segment optical coherence tomography (AS-OCT). The EOM insertion and the direction of the muscle fibers were investigated.

**Subjects and methods:**

A total of 97 healthy normal subjects (194 eyes) at Okayama University Hospital (age, 47.1±21.5 years; range, 8–79 years) participated in the study. A series of 256 tomographic images of the rectus EOMs were captured using the C-scan function of the AS-OCT (CASIA2, TOMEY Co., Japan), and the images were converted to en-face images in multi-TIFF format. The anterior chamber angle to EOM insertion distance (AID) and the angle of the muscle fibers from the insertion site (angle of muscles) were measured from the images. The correlations of AID and angle of muscles with age and axial length were investigated and evaluated.

**Results:**

AID and angle of muscles were significantly correlated with age or axial length in some EOMs. The AIDs of medial rectus (MR) (*P* = 0.000) and superior rectus (SR) (*P* = 0.005) shortened with age. The AIDs of MR (*P* = 0.001) and inferior rectus (IR) (*P* = 0.035) elongated with axial length, whereas lateral rectus (LR) (*P* = 0.013) shortened. The angles of MR (*P* = 0.001) and LR (*P* = 0.000) were found to have a more downward direction toward the posterior in older subjects.

**Conclusion:**

En-face images can be created by AS-OCT, and the shape of the EOMs in normal subjects using these image measurements was available. With the ability to assess the EOMs, AID and angle of muscles are expected give useful information for treating and diagnosing strabismus-related diseases.

## Introduction

Recent advances in anterior segment optical coherence tomography (AS-OCT) have made it possible to visualize not only the cornea, anterior chamber, and iris but also the extraocular muscles (EOMs) [1–9]. However, these reports only involved the evaluation of tomographic images of the EOMs, and most of them were related to muscle insertion. In elucidating the detailed pathology and diagnosis of strabismus, more detailed analysis of the shape of the EOMs may be useful, and the development is expected. There have been reports on the en-face images that superimpose tomographic images to evaluate the shape of the retina, as if each layer were viewed from above, but there have been no reports on the en-face images of the EOMs. The muscle insertion and the direction of muscle fibers can be quickly and easily evaluated using the en-face images depicting the EOMs without the need for ultrasound or direct measurements.

There have been several reports on the insertion site of the EOMs measured from tomographic images of the EOMs using AS-OCT; however, the EOM may be attached obliquely to the corneal rim or in a straight or arched shape, and the location of the tomographic image in the width of the muscle is unknown; hence, measuring the insertion site accurately using a single tomographic image is impossible. The en-face images may be used to measure the insertion site of muscle with respect to the center of the muscle width. In addition, magnetic resonance imaging (MRI) studies of the periphery of the eye have revealed that aging causes loosening, thinning, and tearing of connective tissue such as the periocular pulley and, in particular, downward deviation of the lateral rectus (LR) muscle [10–12], resulting in strabismus, which is no longer an uncommon condition with the increase in the elderly population [13]. Therefore, the direction of muscle fibers from the insertion site of the EOM may be important for pathogenesis and diagnosis and may change with age and eye size, but no findings have been analyzed using AS-OCT.

### Purpose

We attempt to create the en-face images using AS-OCT and evaluate whether the shape of the EOMs in normal subjects can be analyzed. The EOM insertion and the direction of the muscle fibers were analyzed, and the relationship between aging and the axial length was investigated and evaluated.

### Subjects and methods

A total of 97 healthy normal subjects at Okayama University Hospital (age, 47.1±21.5 years; range, 8–79 years) participated in the study. A total of 194 eyes were studied bilaterally. Participants were excluded from the analysis if they had strabismus or history of previous strabismus surgery, if informed consent could not be obtained, or if there was a history of other diseases that cause ocular deviation (e.g., thyroid ophthalmopathy, myasthenia gravis, internuclear ophthalmoplegia, high-grade (pathologic) myopia [14], sensory strabismus, or other neurologic disorders).

**Fig 1:**
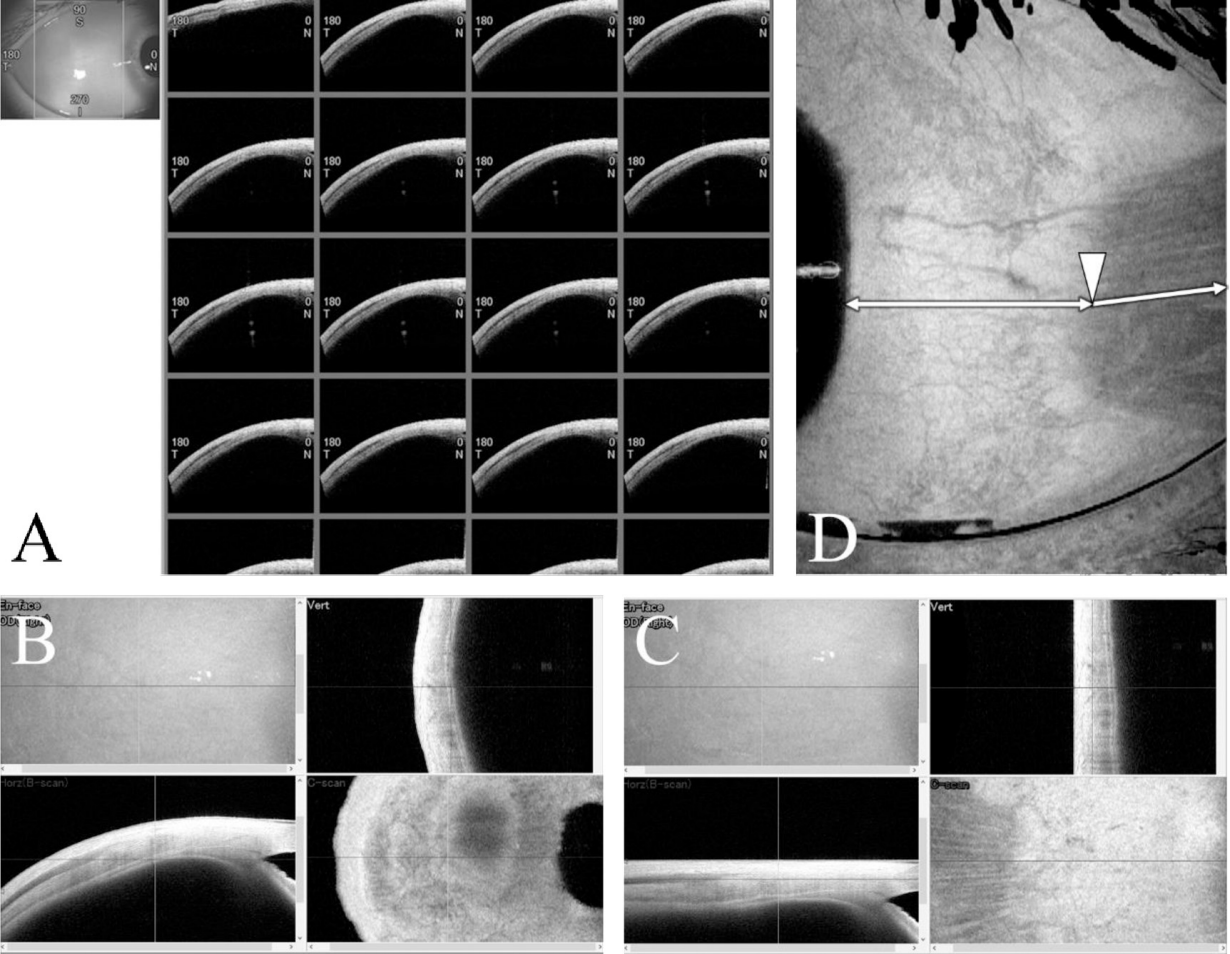
Images rendered by CASIA2, roctia, and ImageJ. **A,** Some of 256 sequential images of tomographic images of EOM. **B,** Before the fluttering process. **C,** After the fluttering processing. **D,** En-face-enhanced image with clearly delineated EOMs and the rim of the anterior chamber angle. *White arrowhead*, midpoint of the muscle width of the EOM; *white double-ended arrows*, shortest distance from the white arrowhead to the rim of the anterior chamber angle; *white arrow*, muscle fiber direction.

The subject was asked to rotate the right eye and the left eye vertically upward and downward and to rotate horizontally inward and outward, respectively, and a series of 256 tomographic images of the medial rectus (MR), LR, superior rectus (SR), and inferior rectus (IR) muscles within a range of 12 mm from the corneal limbus to the direction of the target rectus muscle and 16 mm in width were captured using the C-scan function of the AS-OCT (CASIA2, TOMEY Co., Japan) (Fig 1A). Regarding the measuring eye, the fingertips of the index finger of the subject were placed on the horizontal plane in the left-right direction and on the sagittal plane in the vertical direction, and the subject was instructed to look at the fingertips as much as possible and not move. Fluttering processing was performed using dedicated analysis software roctia (TOMEY Co., Japan), and the images were converted to en-face images in multi-TIFF format (Fig 1B and 1C). Using Python 3.8 (www.python.org, USA), the EOM and the rim of the anterior chamber angle were made into a visualized image (Fig 1D). Using ImageJ 1.52a (Wayne Rasband, National Institute of Health, USA), the minimum distance from the center of the insertion site of the EOM to the rim of the anterior chamber angle (the anterior chamber angle to EOM insertion distance [AID]) and the angle of the muscle fibers from the insertion site of the midpoint of the muscle width to the backward direction (angle of muscles) were measured. The internal rotation side was set to positive and the external rotation side to negative, and the angle of muscle was set to 0° when the angle of MR or LR was horizontal or the angle of SR or IR was vertical. At the same time, the subject was also measured for axial length.

To evaluate whether AID and angle of muscles are measurable, Spearman’s rank correlation coefficient was used to investigate the correlations of AID and angle of muscles with age and axial length. A scatterplot was made with the parameter, and a regression line and curve were obtained for parameters that were significantly correlated in the EOMs. We also compared AID and angle of muscles separately for group A (<50 years old) and group B (>50 years old).

Informed consent was obtained from the subjects. The study protocol conformed to the Declaration of Helsinki and was approved by the institutional review board of Okayama University Hospital (approval number: K1901-006).

## Results

When the eye moved during the imaging process, the en-face images became wavy and/or blurred, making the position of the muscle and the angle of the muscle fibers unclear and the images unsuitable for this measurement. After the images were taken, the images were analyzed at a later date, so they could not be reshot. Some images that were unclear or in which the muscles were unknown because of eyelid coverage were excluded. However, most of the images were of sufficient quality for the analysis.

**Fig 2:**
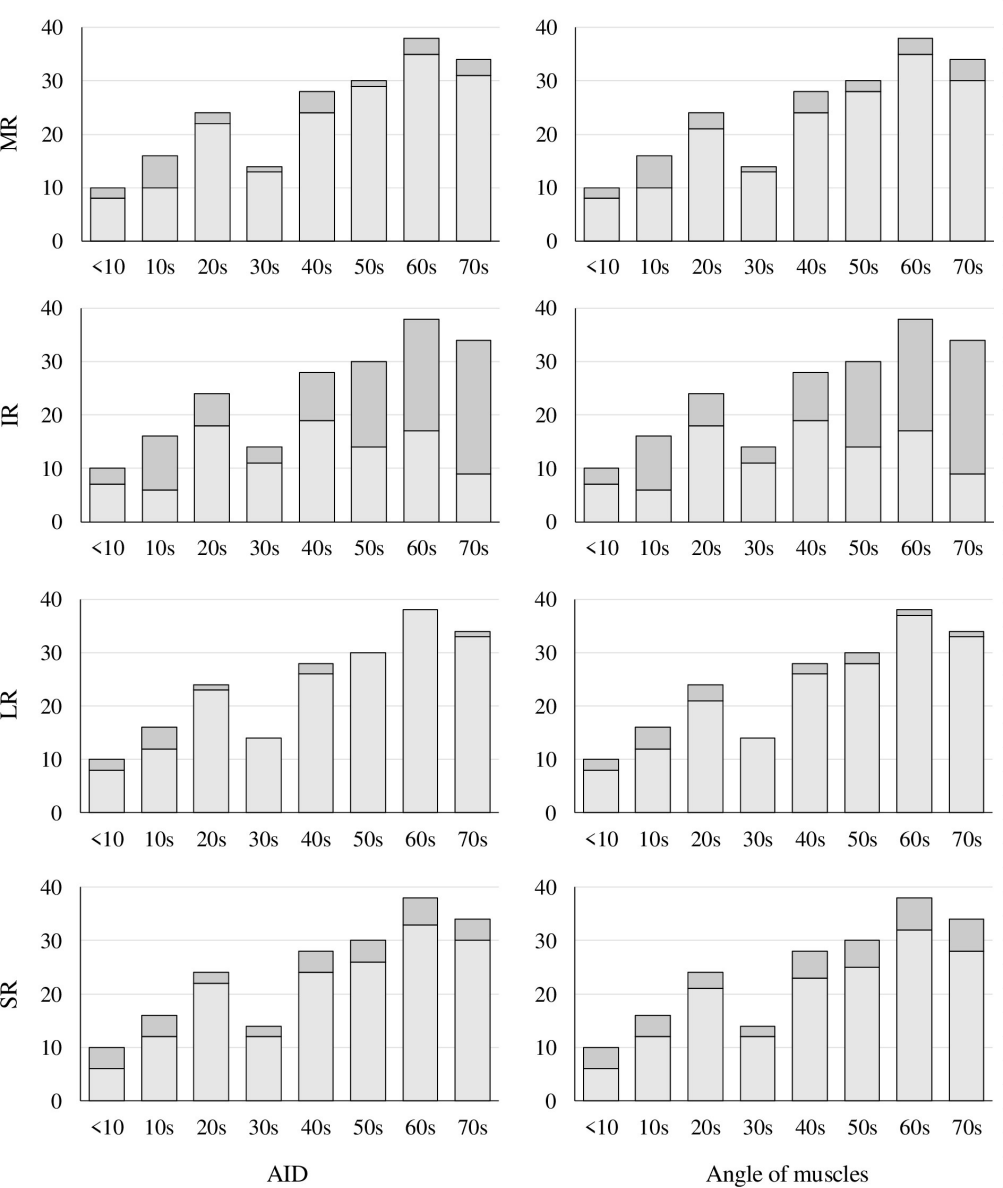
The number of measurable images and exclusions. Stacked histograms of the number of visualized images by age group are shown, with measurable images in light grey and excluded images in dark grey for AID or angle of muscles measurements. They are shown for each rectus EOM.

The results of AID in the MR, IR, LR, and SR are presented in Table 1. The exclusion rate for IR was particularly high. The number of measurable visualized images and exclusions is shown in Fig 2.

**Table 1:** Mean, left-right differences, and exclusion rates for AID and angle of muscles.

	AID (mm)											
	MR			IR			LR			SR		
Left	5.0	±	0.9	4.9	±	0.7	5.9	±	0.6	5.5	±	0.7
Right	5.1	±	0.8	4.8	±	0.7	5.8	±	0.6	5.4	±	0.6
P value	0.249			0.420			0.462			0.620		
Both	5.1	±	0.9	4.8	±	0.7	5.8	±	0.6	5.5	±	0.6
Exclude(%)	11.3			47.9			5.2			14.9		
	Angle of muscles (°)											
	MR			IR			LR			SR		
Left	2.4	±	3.8	-18.2	±	7.7	-2.8	±	4.2	5.7	±	6.2
Right	2.5	±	3.9	-18.0	±	8.4	-2.6	±	4.0	6.4	±	5.8
P value	0.545			0.902			0.759			0.667		
Both	2.4	±	3.8	-18.1	±	8.1	-2.7	±	4.1	6.0	±	6.0
Exclude(%)	12.9			47.9			7.7			18.0		

No significant left-right differences were found in all EOMs. The Mann-Whitney *U* test showed a significance level of *P*<0.05.

The correlations of AID and angle of muscles with age and axial length are presented in Table 2. AID was significantly correlated with age in MR and LR. AID was significantly correlated with ocular axis length in MR, SR, and LR.

**Table 2:** Correlation between the AID and the angle of muscles of the rectus EOMs with respect to age and axial length.

A) Correlation with AID				
	Correlation Coefficient (P value)			
	MR	IR	LR	SR
Age	**-0.306**	-0.043	-0.027	**-0.216**
	(0.000*)	(0.669)	(0.712)	(0.005*)
Axial length	**0.260**	**0.210**	**-0.183**	0.060
	(0.001*)	(0.035*)	(0.013*)	(0.440)
B) Correlation with Angle of Muscles				
	Correlation Coefficient (P value)			
	MR	IR	LR	SR
Age	**0.258**	-0.119	**-0.367**	0.022
	(0.001*)	(0.236)	(0.000*)	(0.781)
Axial length	0.066	0.017	0.019	0.086
	(0.393)	(0.868)	(0.799)	(0.279)

**Fig 3:**
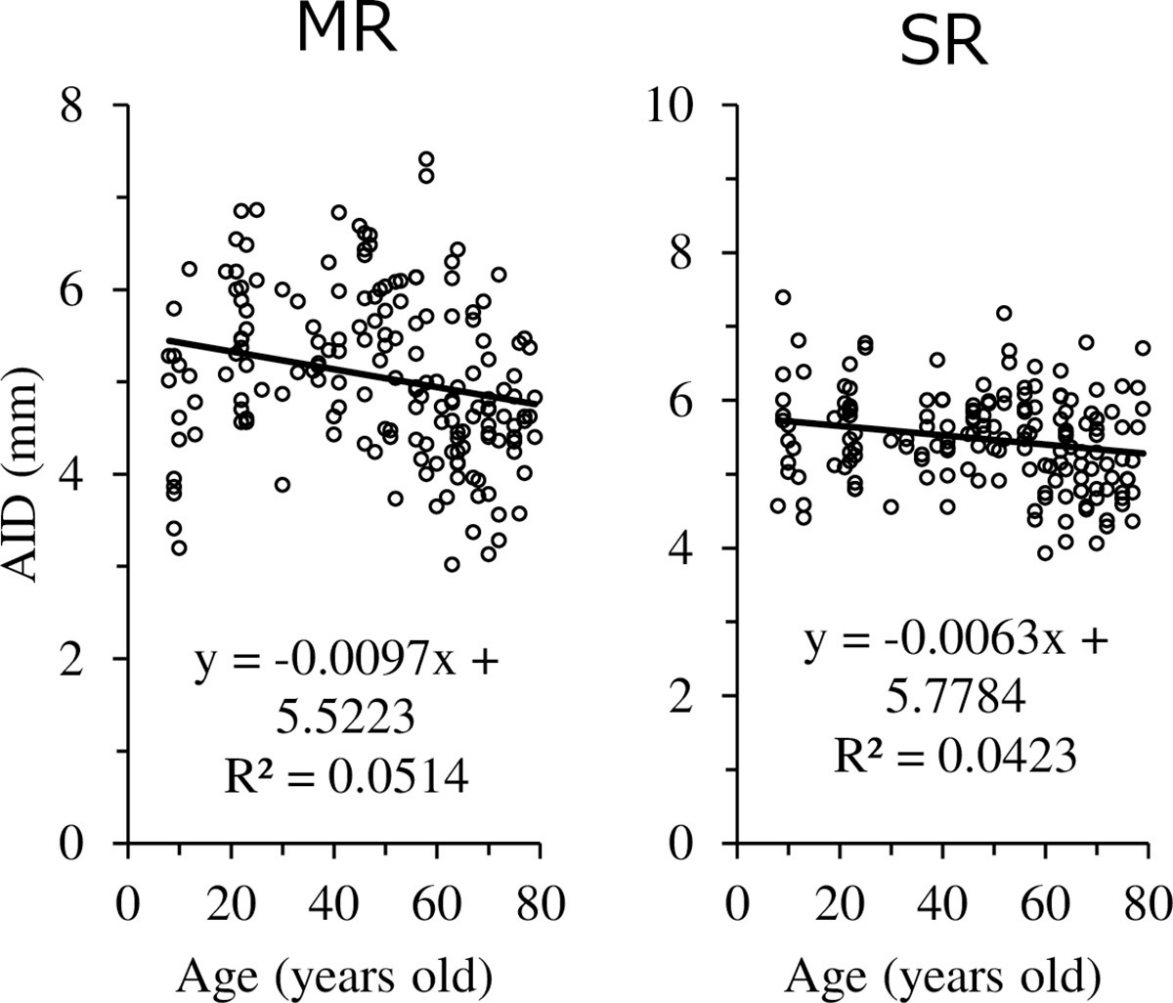
Relationship between age and AID.

The relationship between age and AID is illustrated in Fig 3, where AID shortened with age in MR and SR. The regression curve with age on the X-axis and AID on the Y-axis in MR was also calculated and the following equation (R^2^ = 0.1663) was obtained, which shows the maximum value of AID at age 36.3.

$$y=-0.0008x^{2}+0.0581x+4.3933$$

**Fig 4:**
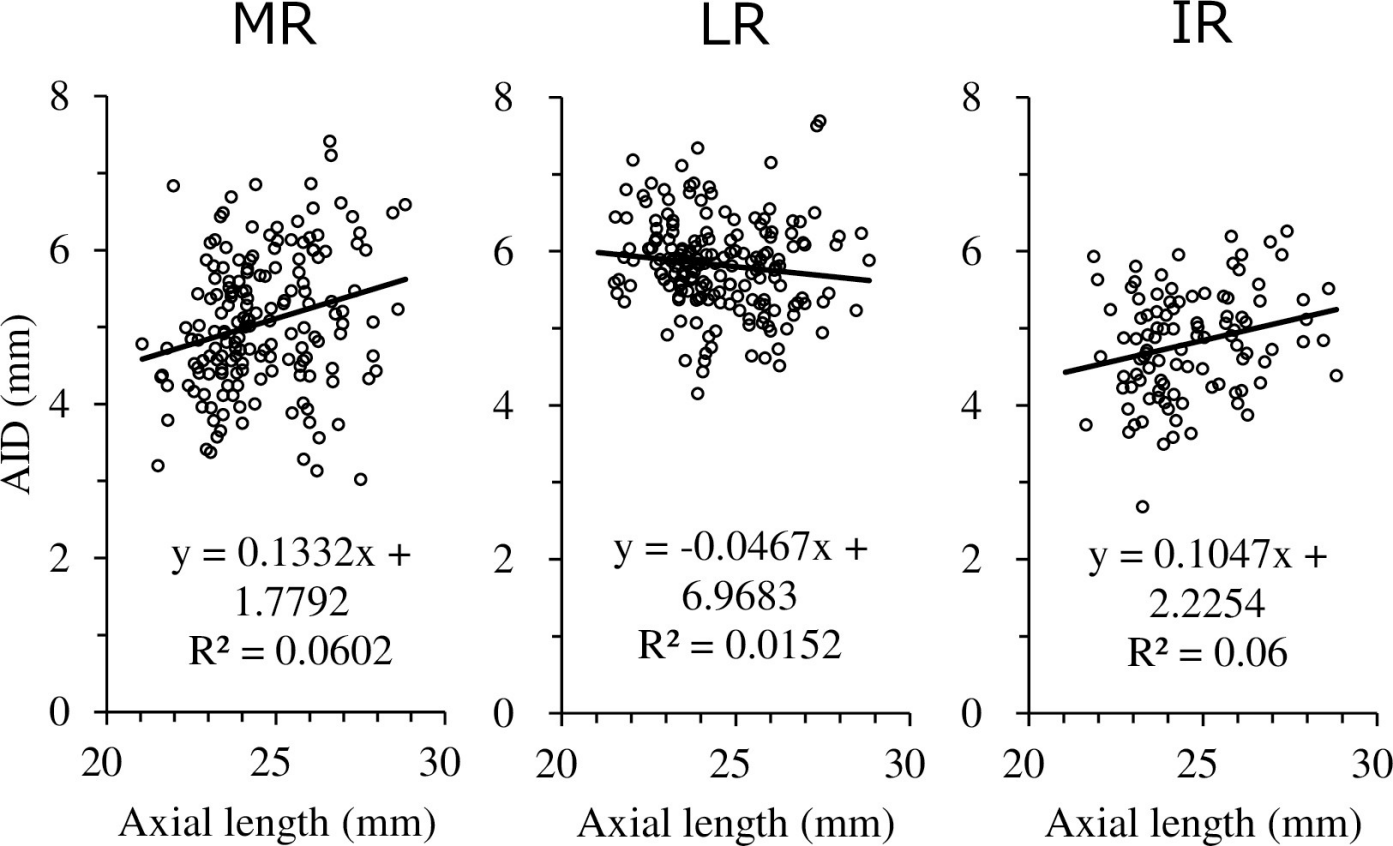
Relationship between axial length and AID.

**Fig 5:**
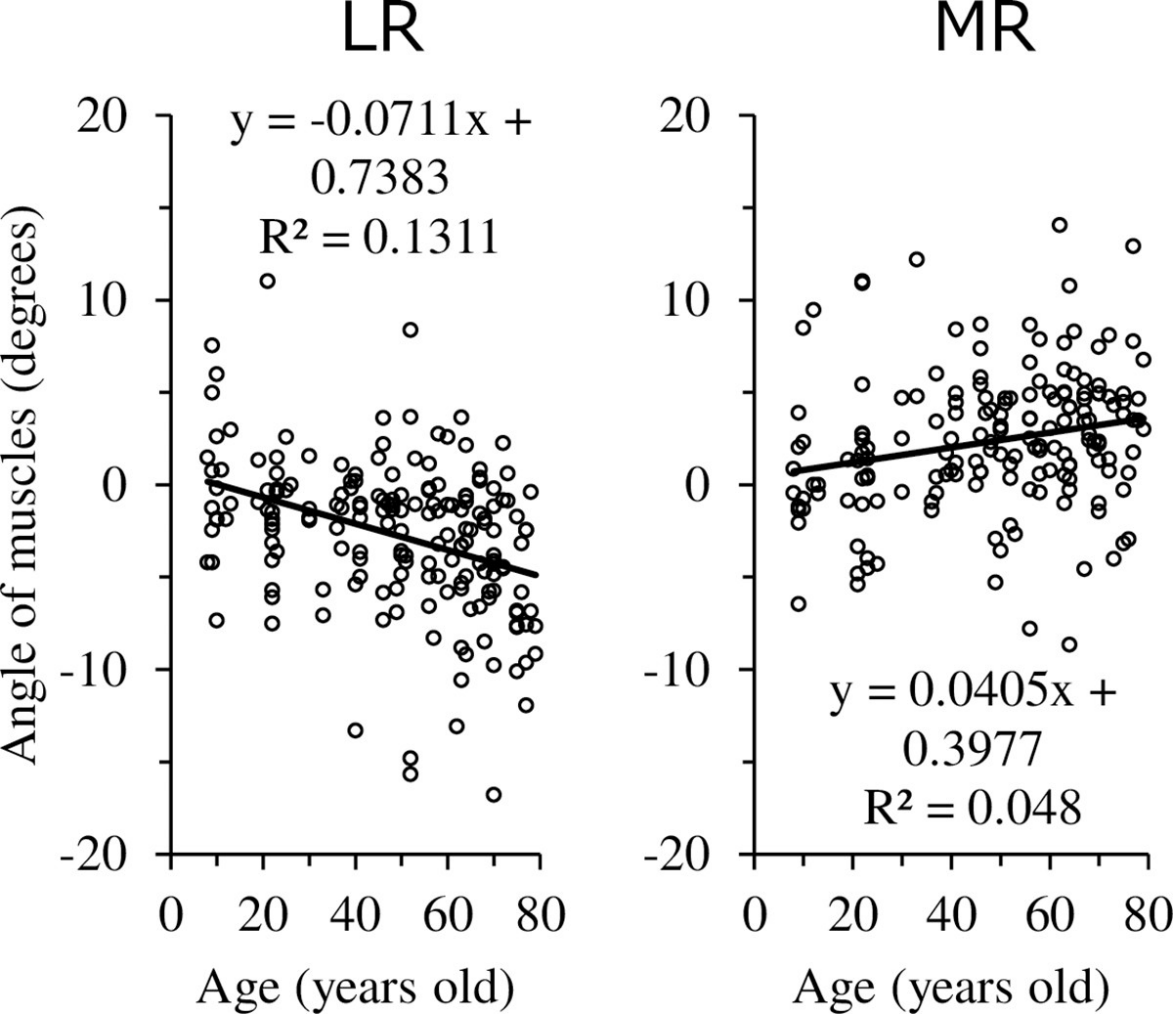
Relationship between age and angle of muscles.

This result indicates that before age 36.3, the AID gets longer with age, but after age 36.3, the AID shortens with age. The relationship between axial length and AID is illustrated in Fig 4. AID was elongated in MR and IR and shortened in LR as the axial length was elongated. The relationship between age and angle of muscles is illustrated in Fig 5. The angle of muscles indicated an increase in internal rotation in MR and an increase in external rotation in LR with age. In other words, MR and LR showed a more downward direction toward the posterior in the areas that could be observed by OCT in older subjects.

AID and angle of muscles by age group are presented in Table 3. Significant differences were also found by group for AID in MR and IR and for angle of muscles in MR and LR.

**Table 3:** Comparison of AID and angle of muscles by age group.

	Group A (<50)	Group B (≥50)	P value
N	46	51	
AID(mm)			
MR	5.4 ± 0.8 (77)	4.8 ± 0.9 (95)	0.000*
IR	4.9 ± 0.6 (61)	4.7 ± 0.8 (40)	0.439
LR	5.8 ± 0.6 (83)	5.8 ± 0.6 (101)	0.671
SR	5.6 ± 0.6 (76)	5.4 ± 0.7 (89)	0.033*
Angle of Muscles(°)			
MR	1.7 ± 3.9 (76)	2.9 ± 3.7 (93)	0.008*
IR	-17.3 ± 8.6 (61)	-19.2 ± 7.1 (40)	0.367
LR	-1.4 ± 3.5 (81)	-3.8 ± 4.3 (98)	0.000*
SR	5.8 ± 6.0 (74)	6.3 ± 6.0 (85)	0.642

Values are mean±standard deviation, and the number of data (n) is indicated in parentheses. Older adults have a higher number of IR images excluded.

*Significant difference, Mann-Whitney *U* test, significance level of *P*<0.05

## Discussion

MR and LR were found to have a more downward direction toward the posterior in older subjects. Clark RA et al. reported that, using MRI, the horizontal rectus EOMs in older subjects were significantly displaced inferiorly throughout the anteroposterior extent of the orbit [15]. Chaudhuri Z et al. also reported sagging eye syndrome, which is an age-related degeneration of the LR-SR bands, allowing the LR pulley to shift and tilt inferolaterally [10]. In this study, the posterior part of the LR in the area of the AS-OCT images is deviated downward in the healthy older subjects (Fig 5). It is hypothesized that patients with sagging eye syndrome may have similar findings, and using AS-OCT, which is clinically easier to measure than MRI, to assess the direction of myofiber may be useful in diagnosing sagging eye syndrome. However, since this study deals with anterior EOMs, more research is needed to investigate their relationship to posterior EOMs or pulleys.

The horizontal rectus EOMs in older subjects were significantly displaced inferiorly [15]. This inferior displacement may reflect the inferior displacement of the pulley corresponding to the EOMs, and part of the vector of the horizontal EOMs’ abduction is converted to a downward vector. In other words, this is a contributing factor to the limited elevation of the eye observed in older subjects. This affected the measurement of IR in AS-OCT, leading to difficulty in determining IR because of difficult elevation of the eye, especially in older subjects (IR exclusion rates, 35% for group A and 60% for group B).

Ocak OB et al. reported a comparison between two groups of normal children aged 8–13 years and normal adults aged 25–30 years and found no statistically significant difference between the two groups in the corneal limbus-EOM insertion distance (LID) of MR but an increase in LID of LR in adults [8]. However, in this study, the AID of MRs tended to shorten with age. According to the regression curve, AID shortened with age after 36.3 years of age. This may be because of differences in study age. Age-related shortening of the AID may be caused by scleral contraction and corner angle recession caused by age-related changes, but further investigation is needed.

In this study, MR and IR AIDs tended to extend with the long eye axis, whereas LRs tended to shorten. Dhakal et al. reported a significantly thinner anterior scleral thickness near the IR muscle in axial myopes. It is suggested that the thinning of the sclera is related to the axial length because of asymmetric scleral expansion. This may be the reason for the extension of IR AIDs.

The distance between the cornea and the insertion site of the rectus EOM was similar to that reported previously (Table 4) [16–23]. However, Indians have a longer average of 8.7 mm of LR and 7.3 mm of MR in the direct measurements [19]. In the AS-OCT reports, Indians also have a longer average than other races [17]. AID may vary by race. AID in all the rectus EOMs was shorter than previously reported. Direct anatomical measurements revealed that the fascial and tenon tissues were attached and the corneal limbus was obscured, which may have displaced the measurement position and made it longer. We considered that only the muscle tissue was measured and the results were different because AS-OCT depicts fascia, tendon, and tenon tissue with a high signal and muscle tissue with a low signal. The distance is slightly different between the corneal limbus and the rim of the anterior chamber angle to be measured, and the distance is shorter for the method of measurement using the rim of the anterior chamber angle. Another reason is that the sphere flutters during the analysis, which shortens the measurement values. However, this measurement error is considered to be small.

**Table 4:** EOM insertion sites in this study and comparison with previous reports.

				Insertion (mm)											
	Nationality	N	Measurement type	MR			IR			LR			SR		
This study	Japanese	97	AS-OCT	5.1	±	0.9	4.8	±	0.7	5.9	±	0.6	5.5	±	0.7
Liu X et al. (2010)	Chinese	37	AS-OCT	5.7	±	0.6				6.8	±	0.6			
Ocak OB et al. (2019)	Turkey (children)	60	AS-OCT	5.7	±	0.8				6.7	±	1.1			
	Turkey (adults)	60	AS-OCT	5.7	±	0.8				6.8	±	1.2			
John J et al. (2017)	Indian	35	AS-OCT	5.8	±	0.2				7.2	±	0.2			
Surawatsatien N et al. (2017)	Thai	46	Direct (dissection)	5.7	±	0.4	6.6	±	0.3	6.9	±	0.5	7.4	±	0.4
Athavale S et al. (2015)	Indian	40	Direct (dissection)	7.3	±	1.6	8.1	±	2.1	8.7	±	2.5	8.7	±	1.7
Villarreal-Silva E et al. (2013)	Mexican	20	Direct (dissection)	6.0	±	0.8	6.8	±	1.0	6.8	±	0.6	7.5	±	1.1
Lai YH et al. (2012)	Chinese	60	Direct (intra-op)	5.2	±	0.9	6.0	±	0.8	6.3	±	0.9	6.8	±	0.7
Tamburrelli C et al. (2003)		19	Ultrasound	5.6	±	0.6				5.8	±	0.6			
		19	Direct (intra-op)	5.5	±	0.8				6.3	±	0.5			
Von Norden's textbook			Direct (dissection) from anterior limbus	5.3	±	0.7	6.8	±	0.8	6.9	±	0.7	7.9	±	0.6
			Direct (dissection) from posterior limbus	4.7	±	0.6	5.9	±	0.8	6.3	±	0.6	6.7	±	0.6

As a limitation, because the eye with an intraocular lens was included, the evaluation was based on the axial length, instead of the equivalent spherical value. Images that cannot be measured because the EOMs cannot be well delineated are excluded. This may exclude cases where the insertion site is more posteriorly located. In particular, IR is unsuitable for measuring using AS-OCT, and the AID of IR may have been shortened.

## Conclusion

En-face images can be created by AS-OCT, and the shape of the EOMs in normal subjects using these image measurements was available. The AIDs of MR and SR shortened with age. The AIDs of MR and IR elongated with axial length, whereas LR shortened. MR and LR were found to have a more downward direction toward the posterior in older subjects. With the ability to assess the EOMs, AID and angle of muscles are expected to give useful information for treating and diagnosing strabismus-related diseases.

## Supporting information

S1 Data (XLSX)
